# Serum electrolyte imbalance and prognostic factors of postoperative death in adult traumatic brain injury patients: A prospective cohort study

**DOI:** 10.1097/MD.0000000000013081

**Published:** 2018-11-09

**Authors:** Pathomporn Pin-on, Ananchanok Saringkarinkul, Yodying Punjasawadwong, Srisuluck Kacha, Drusakorn Wilairat

**Affiliations:** Department of Anesthesiology, Faculty of Medicine, Chiang Mai University, Chiang Mai, Thailand.

**Keywords:** death, electrolyte imbalance, traumatic brain injury

## Abstract

Electrolyte imbalances are common in traumatic brain injury. It shares the cause of perioperative morbidity and mortality. Types of intravenous fluid resuscitation, osmotic diuretics, massive blood loss, and intracranial pathology were considered as the potential factors to worsen electrolyte abnormalities in these patients. The aims of this study were to report the incidence of electrolyte imbalance in traumatic brain injured patients and to assess the association between electrolyte imbalance and other prognostic factors to death within 24 hours of the injury.

The study was carried out in the northern university, tertiary-care hospital of Thailand. The patients aged from 18 to 65 years old, presented with traumatic brain injury, and needed for emergency craniotomy were included. We excluded the patients who had minor neurosurgical procedures, pregnancy, and undergone cardiopulmonary resuscitation from the Emergency Department.

Among 145 patients recruited, 101 (70%) had Glasgow Coma Scale (GCS) score ≤ 8, 25 (17%) had GCS score 9 to 12, and 19 (13%) had GCS score 13 to 15. The most common diagnosis were subdural hematoma and epidural hematoma, 51% and 36%, respectively. Hypokalemia was the most common electrolyte imbalance at 65.5%. The results of the use of a multivariable logistic regression model show that the odds of postoperative death in TBI patients were increased with high levels of blood glucose, hypernatremia, and acidosis.

Hypokalemia was the most common electrolyte imbalance in TBI patients. Hypernatremia, acidosis, and hyperglycemia significantly increased the odds ratio of death in the first 24 hours post TBI.

## 1 Introduction

The World Health Organization (WHO) reported that Thailand had the second highest road fatality rate in the world.^[1]^ Traffic accidents were the primary cause of death in the 15–29-year-old population.^[2]^ Over 70% of all traffic accidents pertain to motorbikes.^[1,2]^ These accidents are the major cause of traumatic brain injury (TBI). Survivors who exist in a vegetative state as a result of traffic accidents need prolonged hospital care and are a significant socio-economic problem in Thailand and other developing countries.^[3–5]^

Electrolyte imbalance in cases of TBI is associated with the pathology of the brain itself or is iatrogenic in causation. Unknown pre-existing conditions such as renal failure, cirrhosis, or congestive heart failure share the cause. It is common and considered as one of several preventable secondary injuries. The risk to the development of electrolyte disturbance in TBI patients depends on the severity of head injury, underlying disease, age, and primary therapeutic strategy such as the choice of resuscitation fluid, administration of mannitol or diuretics, and hyperventilation.^[6–9]^ Of all serum electrolytes, the most common electrolyte subject to imbalance in TBI patients is serum sodium.^[5,10–12]^ Suman et al^[5]^ and Rafiq et al^[10]^ reported the most common electrolyte imbalance condition in TBI was hypernatremia followed by hyponatremia and hypokalemia. Rhoney et al^[6]^ reported hyponatremia as being a common electrolyte disturbance in cases of TBI, which was most often caused by a disorder known as the syndrome of inappropriate antidiuretic hormone (SIADH). Transient hypothalamic-pituitary adrenal (HPA) dysfunction and secondary adrenal insufficiency (AI) were reported in moderate to severe TBI patients and these conditions may cause sodium disturbance.^[13,14]^ Many previous studies have reported variations in the incidence and type of electrolyte imbalance in TBI patients but not in association with the morbidity and mortality.

This study aimed to report on the incidence of electrolyte imbalance in adult TBI patients and to assess the association between hyper-acute, un-corrected, and theoretically short-lived electrolyte abnormalities and early outcomes focusing on postoperative death in the first 24 hours. The secondary aim was to identify the prognostic factors of postoperative mortality in traumatic brain injured patients.

## 2 Methods

We hypothesized that un-corrected preoperative electrolyte disorders are associated with perioperative mortality. A prospective cohort study was conducted with the approval of the Institutional Ethical Committee of Faculty of Medicine, Chiang Mai University (CMU), Thailand. All TBI patients admitted during the period January 2016 to February 2017 were included in the study. The informed consent had been obtained from all participants. The inclusion criteria were patients in the age group of 18 to 65 years who were scheduled for emergency craniotomy or craniectomy. Exclusion criteria were minor neurosurgical procedures (e.g., ventriculostomy or burr hole), pregnancy, and TBI patients who had undergone cardiopulmonary resuscitation (CPR) from the Emergency Department (ED). A total of 145 patients were enrolled onto the study.

Patients were assessed using the Glasgow Coma Scale (GCS) on the first arrival at the ED to indicate the severity of brain injury. A GCS score ≤ 8 means severe TBI, 9 to 12 means moderate TBI, and 13 to 15 means mild TBI. Laboratory investigations were completed on all patients before and after surgery including complete blood count (CBC) and serum electrolyte levels. Normal values of blood serum chemistry were quoted from the investigations operations manual 2015.^[15]^ Hyponatremia was defined as serum sodium equal to or less than 130 mEq/L; serum sodium of 131 to 145 mEq/dL was classified as the normal range and serum sodium of 146 mEq/L or greater as hypernatremia. Hypokalemia was defined as a serum potassium level of <3.5 mEq/L and a serum potassium level of >5.0mEq/L as hyperkalemia. Serum calcium levels of <8.0 mg/dL and more than 10.0 mg/dL were defined as hypocalcemia and hypercalcemia. Serum phosphate levels of <3.5 mg/dL and >5.5 mg/dL were defined as hypophosphatemia and hyperphosphatemia respectively. Normal serum magnesium was taken as 1.5 to 3.0 mEq/l and normal serum bicarbonate as 22 to 26 mmol/L.

All patients had general anesthesia by the total intravenous anesthetic technique (TIVA) protocol for TBI. An arterial catheter was applied to all patients for real time blood pressure monitoring and to enable arterial blood gas assessment. Type of intravenous fluid, operation time, blood loss, and blood component administration were recorded. The patients were followed up to 24 hours after surgery. The occurrence of postoperative death was recorded.

The primary outcome of this study was the incidence of electrolyte disorder in TBI patients. A descriptive statistics were used to express the results. The secondary outcomes were analyzed by using univariate and multivariate logistic regression statistics. Univariate was used to select independent variables for multivariate analysis. Each independent variable was entered into the logistic model in a stepwise fashion. The logistic regression model was built for risk factors of mortality rate within 24 hours post-injury. We interpreted a *P*-value < .05 as significant at a 95% confidence interval (CI). Data entry and statistical testing were done using the Statistical Package for STATA version 14.2. There was no loss-to-follow up case because all patients were admitted in the neuro-intensive care unit and were being follow-up for outcome in 24 hours post-injury.

## 3 Results

In the period January 2016 to February 2017, 168 patients fulfilled the selection criteria. In 17 cases it was not possible to obtain informed consent and 6 patients suffered cardiac arrest during the craniotomy. The remaining 145 adult patients were enrolled in the present study. 70% of them were classed as severe TBI. The characteristics are presented in Table 1. Postoperative death in 24 hours occurred in 25 patients (17.2%). All patients were classified as severe TBI. As can be seen from Table 1, TBI occurred more commonly in men than in women. The distribution of data from the American Society of Anesthesiologists (ASA) classification was greater in class III as a result of associated injuries. Subdural and epidural hematomas were common findings in TBI patients who required emergency craniotomy.

**Table 1:**
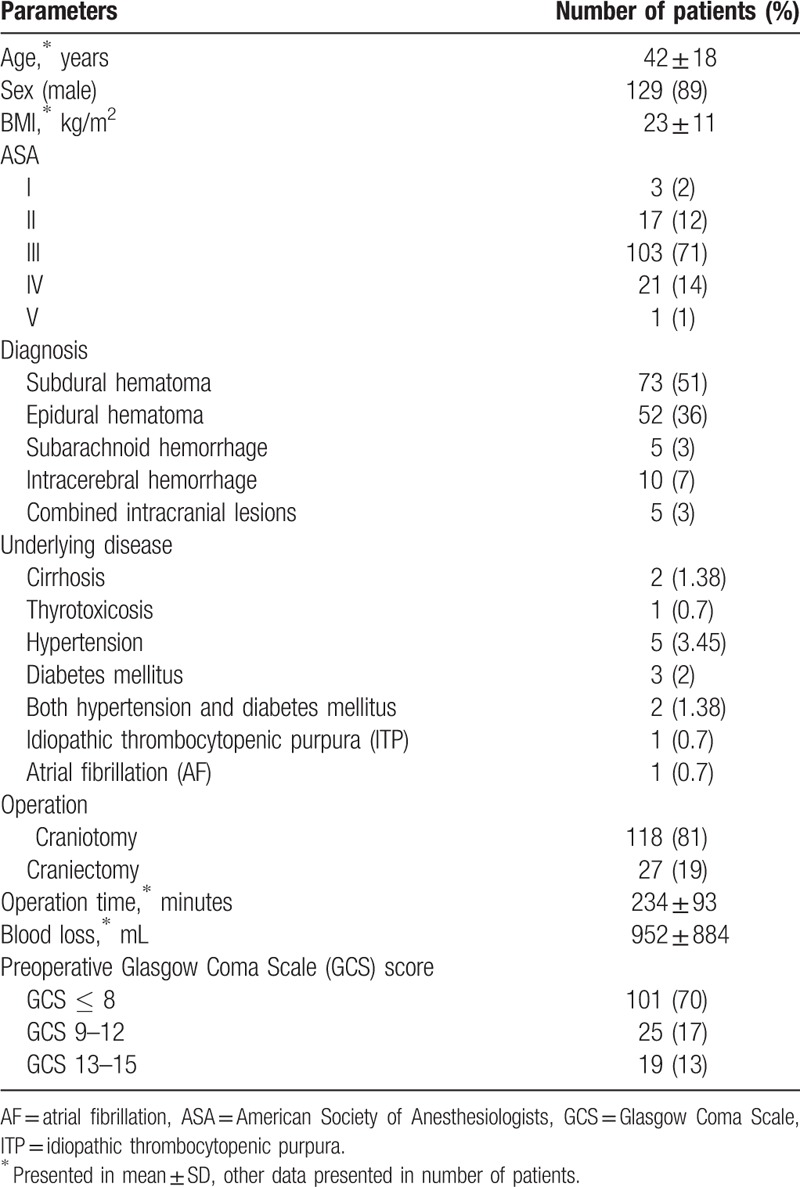
Demographic data.

Table 2 shows the incidence of electrolyte imbalance in perioperative period. Hypernatremia was found more common than hyponatremia. 86.3% of hypernatremic patients were in the severe TBI group. Around 76.7% of hypernatremic patients died in the first 24 hours after surgery. The occurrence of hypernatremia was highest in patients diagnosed with subarachnoid hemorrhage (SAH). We found that hypernatremia occurred more often in the post-surgery period than preoperative period whilst hyponatremia was found to be similar in the preoperative and postoperative periods. Hypokalemia was the most common electrolyte imbalance in preoperative period (65.5%). The average value of preoperative hypokalemia was 3.17 ± 0.55 mEq/L. Potassium infusion during surgery and serial arterial blood gas were the main reasons of decreasing the incidence of postoperative hypokalemia. Calcium disturbance was markedly increased in postoperative period. Phosphate and magnesium depletion were remarkably high in severe TBI patients. Combined metabolic and respiratory acidosis was the most common electrolyte imbalance in postoperative period (67.5%).

**Table 2:**
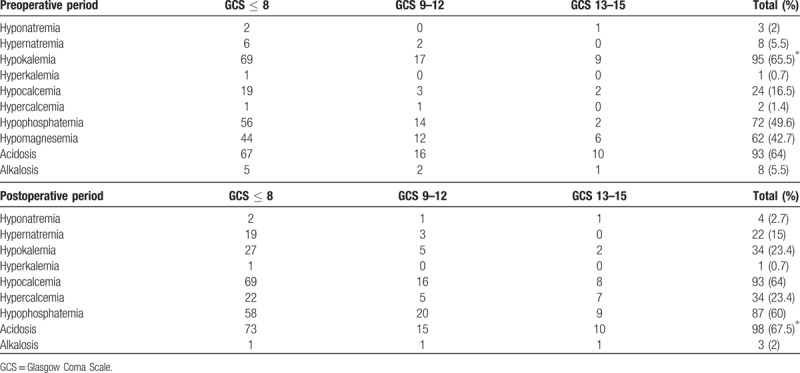
Incidence of electrolyte imbalance in preoperative and postoperative period.

Table 3 shows the variations and average volume of intravenous fluid and blood components. There was no hypertonic saline solution used in order to reduce intracranial pressure. Mannitol was used in only 8 patients due to medical concerns regarding hemodynamic instability. Around 82 out of 101 patients (81.2%) in the severe TBI group, 11 out of 25 patients (44%) in the moderate TBI group, and 3 out of 19 patients (15.8%) in the mild TBI group required packed red cells transfusion. The ratios of fresh frozen plasma transfusion were 55.4%, 24%, and 15.8%, in the severe, moderate, and mild TBI groups, respectively. Platelet transfusions were 34.6%, 8%, and 5.3%, respectively.

**Table 3:**
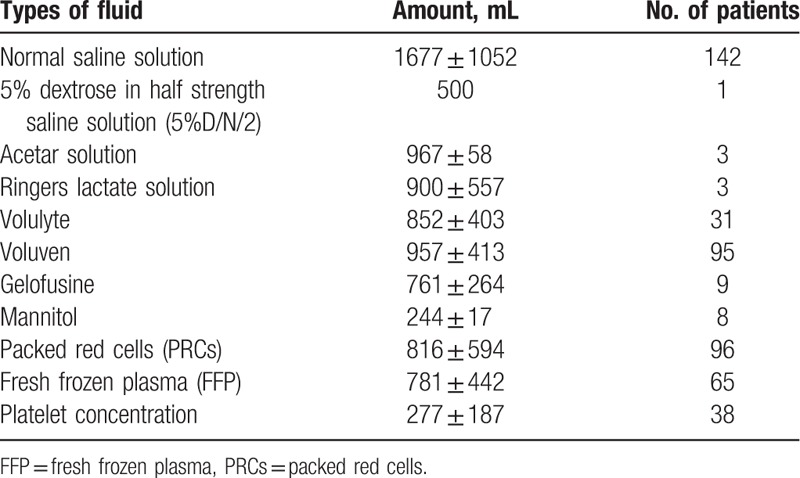
Intravenous fluid and blood component administration in cases of traumatic brain injury.

**Figure 1:**
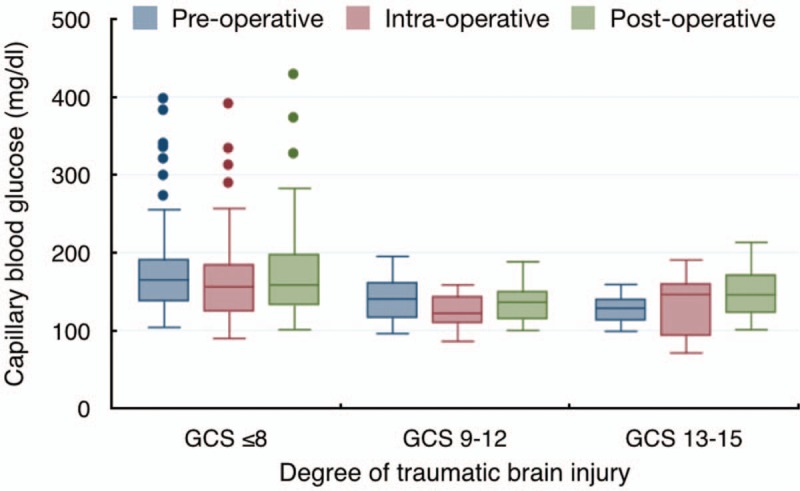
Blood glucose variation according to the severity of TBI. GCS = Glasgow Coma Scale, TBI = traumatic brain injury.

Table 4 shows the difference in perioperative prognostic factors between the 3 groups. Intentional hyperventilation was used universally. Current published data show profound hypocapnia in severe TBI patients. Significantly greater blood loss meant the patients required greater input of red cells and fresh frozen plasma transfusion in severe TBI patients. Blood glucose was also considerably higher with several outliers in severe TBI cases (Fig. 1).

**Table 4:**
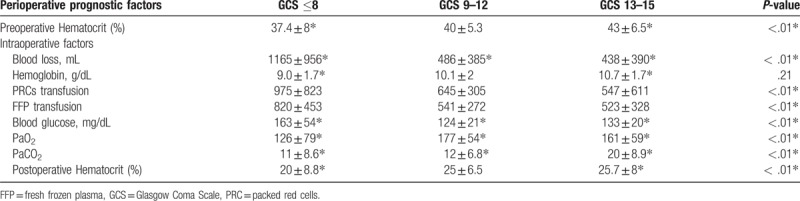
The predicted prognostic factors of each group of patients with traumatic brain injury.

The results of the use of a multivariable logistic regression model are shown in Tables 5 and 6. The results show that the odds of postoperative death in TBI patients were increased with high levels of blood glucose, hypernatremia and acidosis.

**Table 5:**
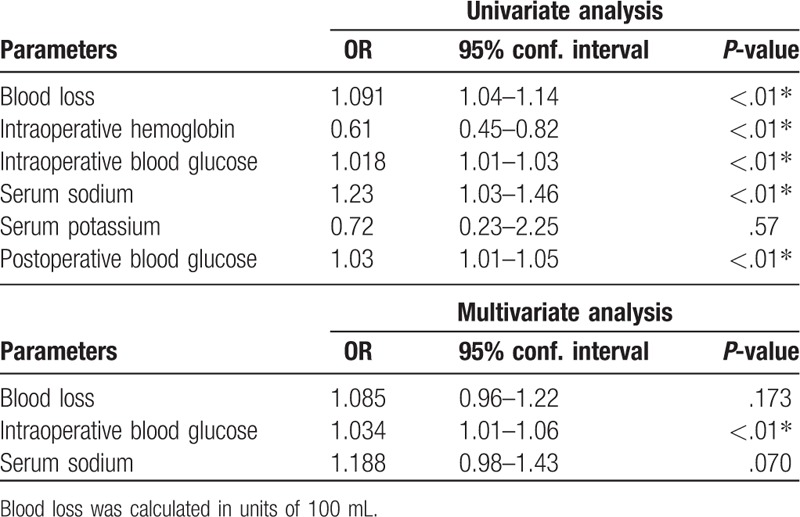
Odds ratio of postoperative death within 24 hours of traumatic brain injury.

**Table 6:**
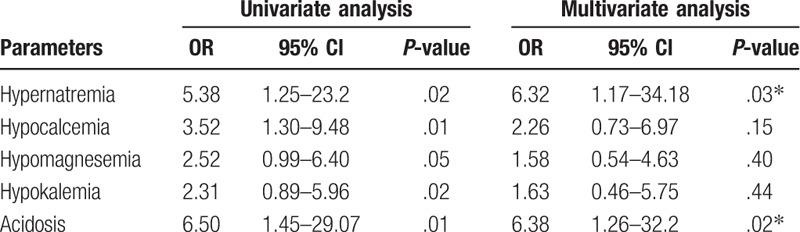
Odds ratio of electrolyte imbalance and postoperative death within 24 hours of traumatic brain injury.

## 4 Discussion

Contrary to many previous reports, in this study it was found that hypokalemia was the most commonly found electrolyte imbalance in TBI patients. In postoperative period, acidosis was the most common electrolyte imbalance. Even though serum sodium disturbance was not found to be the most common electrolyte imbalance, hypernatremia significantly increased the odds of postoperative death in the first 24 hours after head injury. Acidosis and increased blood glucose were the most important predictive factors of death in TBI patients. Serum blood glucose was significantly greater with several high outliers in severe TBI patients. One possible explanation for this was the uncertainty around the time of their last meal prior to the injury whilst TBI patients who had a greater level of consciousness in the moderate and mild TBI groups could give more details about the time of their last meal. The abnormal preoperative electrolyte values were not corrected as the patients needed an emergency surgery. The corrective strategy was applied during the surgery. The findings of the present study supported the hypothesis that the incidence of electrolyte imbalance was affected by the severity of TBI. However, further multicenter trials are needed to add weight to these findings and to affirm that this is a causal relationship. The clinical significance of the findings are twofold. One is the study highlights the need to increase the level of surveillance of patients to ensure common electrolyte imbalances in TBI, such as hypokalemia, are identified quickly and adjustments are made. Secondly predictive factors of death in TBI patients, such as hypernatremia, acidosis, and hyperglycemia are identified and corrected quickly. The emergency setting of this study limits the completeness of individual patient's pre-existing conditions that might affect the electrolyte status such as renal impairment, severity of cirrhosis, or congestive heart failure.

Previous studies found that the most powerful independent variables associated with TBI outcomes were age, GCS motor score, pupil response, Marshall CT classification and traumatic subarachnoid hemorrhage.^[16,17]^ This supports the finding from previous studies that a lower GCS on admission was a strong prognostic factor of a worse outcome. As the data shows all deceased patients were in the severe TBI group.^[17–19]^ However, one limitation of this study was the lack of results regarding the association between vital signs, age, and CT findings and mortality rate due to the greater focus on laboratory parameters and selective intraoperative factors.

The results from the IMPACT study demonstrated that the prognostic values of serum sodium and Glasgow Outcome Score (GOS) are shown in a U-shape. This means that hyponatremia was strongly related to a poorer outcome.^[20]^ Maggiore et al reported that hypernatremia was associated with a threefold increase in an adjusted hazard ratio of ICU death in patients with severe traumatic brain injury.^[21]^ Similarly, Li et al^[22]^ reported severe hypernatremia as being an independent risk factor with an extremely high odds ratio for death in TBI patients who were admitted to the neurosurgical intensive care unit (NICU). The current findings support these previous studies however they did contrast to the results from the IMPACT study. A limitation of our study was a deficiency of information regarding the cause of hypernatremia. Additional data such as urine output, urine specific gravity, and vasopressin (DDAVP) administration is required in order to verify the presence or absence of central diabetes insipidus (CDI). CDI is considered as an important marker of the extension and severity of brain injury.^[22–25]^ An additional study partially agrees with the present findings stating that hypernatremia in severe TBI patients was associated with an increased risk of death, and a longer ICU stay.^[26]^ The authors indicated that the association between hypernatremia and death in TBI patients was independent of GCS.^[26]^ Contrary to the present study, the results of that study shows a strong correlation between hypernatremia, low GCS, and postoperative death in TBI patients.

Hyperosmolar therapy, including mannitol or hypertonic saline solution, promotes fluid shifts from interstitial compartments into intravascular compartments and then diuresis. It is established as one of the most effective ICP lowering therapies.^[27]^ In the present study, none of the patients received hypertonic saline solution and only a small proportion (5.5%) were given mannitol. However, all 8 patients who were given mannitol developed hypernatremia postoperatively.

The increase of hypocalcemia in the postoperative period could be the result of blood transfusion and the citrate chelation of serum ionized Ca.^[28,29]^ Citrate is a preservative and anticoagulant typically added in each unit of packed red cells and fresh frozen plasma. Citrate is metabolized by liver enzymes and cleared from the circulation in minutes. However, hypoperfusion and acidotic conditions delay metabolic processes. The present results show that even though severe TBI patients were given significantly more units of blood by transfusion, they were still more anemic in comparison to patients in the mild and moderate TBI groups. Isolated postoperative hypocalcemia was not shown to be a predictive factor of death in TBI patients (*P* = .79). Overall it can be concluded that the combination of inadequate blood transfusion, hypoperfusion, acidosis, and hypocalcemia forms a vicious loop and worsens the outcomes.

The findings of this study regarding hypophosphatemia and hypomagnesemia are similar to those previously reported by Lindsey et al.^[30]^ Those authors concluded that multiple trauma patients with TBI required phosphorus and potassium supplements at a greater level than non-TBI patients. This conclusion is in alignment with the recommended management guidelines for TBI patients which emphasize the importance of early nutritional support to decrease the chances of mortality.^[9]^

## 5 Conclusions

We found that hypokalemia was the most common electrolyte imbalance in TBI patients. Perioperative hypernatremia, acidosis, and increased blood sugar significantly increased the odds ratio of postoperative death in the first 24 hours post TBI.

## Acknowledgment

Special thanks to Dr Kajohnsak Noppakun for the statistical analysis and statistic comments.

## Author contributions

**Conceptualization:** Pathomporn Pin-on, Yodying Punjasawaswong.

**Data curation:** Ananchanok Saringkarinkul, Srisuluck Kacha.

**Formal analysis:** Pathomporn Pin-on.

**Investigation:** Pathomporn Pin-on.

**Methodology:** Pathomporn Pin-on.

**Project administration:** Srisuluck Kacha.

**Resources:** Drusakorn Wilairat, Srisuluck Kacha.

**Validation:** Pathomporn Pin-on, Yodying Punjasawaswong.

**Visualization:** Ananchanok Saringkarinkul, Drusakorn Wilairat.

**Writing – original draft:** Pathomporn Pin-on.

**Writing – review & editing:** Pathomporn Pin-on.

Pathomporn Pin-on: 0000-0001-7371-8433.

Pathomporn Pin-on orcid: 0000-0001-7371-8433.
